# Individual nutrition therapy and exercise regime: A controlled trial of injured, vulnerable elderly (INTERACTIVE trial)

**DOI:** 10.1186/1471-2318-8-4

**Published:** 2008-02-26

**Authors:** Susie K Thomas, Karen J Humphreys, Michelle D Miller, Ian D Cameron, Craig Whitehead, Susan Kurrle, Shylie Mackintosh, Maria Crotty

**Affiliations:** 1 Rehabilitation and Ageing Studies Unit, Flinders University, Adelaide, South Australia, Australia; 2 Department of Nutrition and Dietetics, Flinders University, Adelaide, South Australia, Australia; 3 Rehabilitation Studies Unit, University of Sydney, New South Wales, Australia; 4 Division of Rehabilitation and Aged Care, Hornsby Ku-ring-gai Hospital, Hornsby, New South Wales, Australia; 5 School of Health Sciences, University of South Australia, Adelaide, South Australia, Australia

## Abstract

**Background:**

Proximal femoral fractures are amongst the most devastating consequences of osteoporosis and injurious accidental falls with 25–35% of patients dying in the first year post-fracture. Effective rehabilitation strategies are evolving however, despite established associations between nutrition, mobility, strength and strength-related functional outcomes; there has been only one small study with older adults immediately following fragility fracture where a combination of both exercise and nutrition have been provided. The aim of the INTERACTIVE trial is to establish whether a six month, individualised exercise and nutrition program commencing within fourteen days of surgery for proximal femur fracture, results in clinically and statistically significant improvements in physical function, body composition and quality of life at an acceptable level of cost and resource use and without increasing the burden of caregivers.

**Methods and Design:**

This randomised controlled trial will be performed across two sites, a 500 bed acute hospital in Adelaide, South Australia and a 250 bed acute hospital in Sydney, New South Wales. Four hundred and sixty community-dwelling older adults aged > 70 will be recruited after suffering a proximal femoral fracture and followed into the community over a 12-month period. Participants allocated to the intervention group will receive a six month individualised care plan combining resistance training and nutrition therapy commencing within 14 days post-surgery. Outcomes will be assessed by an individual masked to treatment allocation at six and 12 months. To determine differences between the groups at the primary end-point (six months), ANCOVA or logistic regression will be used with models adjusted according to potential confounders.

**Discussion:**

The INTERACTIVE trial is among the first to combine nutrition and exercise therapy as an early intervention to address the serious consequence of rapid deconditioning and weight loss and subsequent ability to regain pre-morbid function in older patients post proximal femoral fracture. The results of this trial will guide the development of more effective rehabilitation programs, which may ultimately lead to reduced health care costs, and improvements in mobility, independence and quality of life for proximal femoral fracture sufferers.

**Trial registration:**

Australian Clinical Trials Registry: ACTRN12607000017426.

## Background

Proximal femoral fracture (PFF) is amongst the most devastating consequences of osteoporosis and injurious accidental falls with 25–35% of patients dying in the first year post-fracture [1,2], only 40% returning to pre-fracture level of mobility [3], 15% failing to regain sufficient independence to remain in their own home [4] and annual expenditure for fractures in the United States alone exceeding $20 billion [5]. With the world-wide number of hip fractures projected to approximately double to 2.6 million by the year 2025, and 4.5 million by the year 2050, there is an urgent need to develop preventive strategies [6].

For older people who sustain PFF, rapid deconditioning, loss of muscle strength and weight loss often develop. Causes for this may include inadequate dietary intake, hypermetabolism, and a decline in skeletal muscle mass as a result of a complex interaction between immobility, sarcopenia, wasting and possibly cachexia [7,8]. Muscle weakness can lead to impaired balance and slowed gait speed and it is likely that this rapid deconditioning is partly responsible for the hospital readmissions, repeat injurious falls and loss of independence associated with PFF in older people [9-12].

Exercise programs with community-dwelling, medically stable older adults have been extensively evaluated, the findings suggesting significant reductions in falls [13-15], injuries [13,14], activity restrictions [16] and improvements in balance [17,18]. However, only a small number of exercise studies have been undertaken beginning in the early recovery phase after PFF. All have evaluated the effect of a strengthening program incorporating progressive resistance exercise and, while the findings of some studies appear to be positive in terms of improved strength, gait speed, balance and quality of life (QOL) [19-24], others have found no effect on physical performance and an increased risk of subsequent injury [25]. However, many of these studies were undertaken on samples combining PFF patients with other diagnostic groups (e.g. frail, elective, orthopaedic) and hence the impact of an exercise program for PFF patients alone is uncertain. Also, many studies have commenced after rehabilitation when it is likely that there has already been rapid deconditioning and significant decline in skeletal muscle mass and strength.

The quality of evidence supporting nutritional interventions following PFF is limited and inconsistent [26]. One of the main limitations of studies evaluating the effect of nutrition support following PFF is the lack of attention to the provision of an adequate amount of energy and protein to address the deficits associated with pre-existing undernutrition [26], injury and surgical stress [27] and inadequate dietary intakes during recovery [7,8]. Indeed, most nutrition support studies prescribe a standard volume of oral supplement based on a convenient number of tins rather than individual needs [26] and there are no studies that approximate usual clinical care by also including individual goal setting, menu modification and counselling.

Despite established associations and potential interactions between nutrition, mobility, strength and strength-related functional outcomes, there has been only one small study (n = 100) amongst older adults immediately following lower limb fragility fracture where a combination of both exercise and nutrition have been provided [28]. In this study interventions commenced seven days following injury and consisted of either an individually prescribed multi-nutrient energy dense oral supplement for six weeks, tri-weekly resistance training for 12 weeks, combined treatment or attention control. The findings of this small study highlighted for the first time that nutritional management with a concurrent exercise program post lower limb fragility fracture may be able to prevent significant weight loss. Despite clinically significant weight loss across all treatment groups, this study identified that it is possible to achieve weight maintenance if strategies are implemented to ensure the nutrition supplementation prescription is adequate to achieve energy balance and levels of adherence are high. It also suggested that a comprehensive approach to nutrition support, rather than oral supplements alone, is likely to be required to improve nutritional status and hence impact positively on functional outcomes or quality of life (QOL).

The aim of this randomised controlled trial (RCT) is to implement a six month, individualised exercise and nutrition program commencing within 14 days of surgery for PFF, that will result in clinically and statistically significant improvements in terms of physical function, body composition and QOL at an acceptable level of cost and resource use and without increasing the burden of caregivers. Comparisons will be drawn with the attention control group.

## Methods and Design

### Design overview

**Figure 1:**
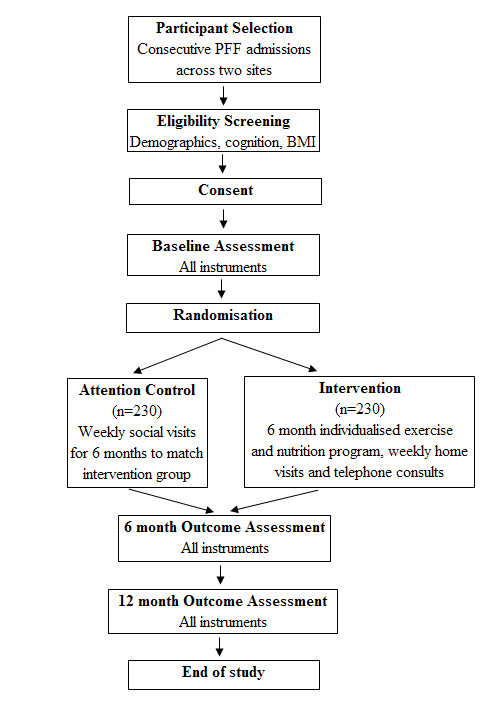
Study design and assessment points.

A two-site RCT with masked outcome assessment and 12 month follow up of 460 community-dwelling older adults post PFF. Participants will be randomly allocated to: a) six month individualised exercise and nutrition program (intervention group) or b) attention control (control group). An overview of the recruitment method, randomisation process and follow-up procedures is provided in Figure 1.

### Recruitment and eligibility

Participants will be recruited from two acute care settings: Flinders Medical Centre (Adelaide), and Hornsby Ku-ring-gai Hospital (Sydney). Eligible individuals will be all those admitted with a diagnosis of PFF confirmed by radiology report, aged 70 years and over and residing in the community, within existing local service boundaries.

Screening will be carried out for all individuals who meet these inclusion criteria by project personnel in consultation with members of the orthogeriatric team. Potential participants will need to achieve a mini-mental score (MMSE) > or = 18/30, as it is thought that participants with severe cognitive impairment [29] will be unable to independently follow therapeutic advice and have difficulty in verification of QOL and other outcomes. If a score of = 18/30 or above is not achieved on the first attempt following surgery and post operative delirium is suspected then the MMSE will be repeated at a later date (but still within 14 days of injury) to see if a MMSE score of = 18/30 or above can be achieved. Participants must also have a body mass index (BMI) between 18.5 kg/m^2 ^and 35 kg/m^2^. A BMI less than 18.5 kg/m^2 ^is considered to be underweight [30] and ethically these patients should receive nutrition support as standard care. Patients with a BMI >35 kg/m^2 ^(morbidly obese) [30] will be excluded from participating in the study as there is currently no evidence that the same level of decline in body weight exists for these patients and these patients are likely to have multiple medical co-morbidities increasing the complexity of the study intervention.

Exclusion criteria include a pathological fracture or malignancy; those residing in residential care, non-English speaking, limited to stand transfers only post surgery or non ambulatory pre fracture, unable to give informed consent or not deemed to be medically stable within fourteen days post surgery. All those individuals who meet the inclusion and exclusion criteria will be invited to participate. When study personnel are satisfied that an individual understands the study information and implications of their involvement, written informed consent will be obtained prior to commencement of baseline measures and randomisation. In the situation where it is felt that a participant does not have the capacity to give informed consent (i.e. post-operative delirium or mild cognitive impairment) third party consent will be obtained from a close relative or immediate caregiver.

The Flinders Clinical Research Ethics Committee reviewed and approved the study protocol – Research Application 110/067.

### Measurements and Procedures

#### Baseline, six and 12 month follow-up

Project personnel will assess the selected outcome measures at baseline (prior to randomisation) whilst an individual masked to treatment allocation will assess outcomes at six and 12 months. Testing will coincide with maximum effect of each participant's analgesic regime. Outcome measures have been selected based on suitability for older adults following PFF, strength of the evidence to support validity and reliability, significance to rehabilitation and previous use in the literature.

The baseline assessment protocol entails a detailed multi-dimensional health assessment by means of:

**a) Physical Function, Strength and Balance: **Physical autonomy will be measured using a physical and instrumental ADL scale [31]. A subjective rating of pain for three separate activities of daily living (ADL) will be established prior to physical testing, which include knee extensor strength using a Nicholas Manual Muscle Tester (NMMT) [32] and a 30 second chair stand test [33], gait speed via a 3 m Walk Test [34], balance on the modified Berg Balance Scale (MoBerg) [35] and grip strength using a hand held dynamometer [36].

**b) Body Composition and Nutrition Measures: **Percent weight change will be assessed using calibrated scales. Body composition (fat free mass) will be measured at baseline, six and 12 months using dual-energy x-ray absorptiometry (DEXA: Lunar Prodigy, GE Healthcare, UK). Lumbar spine and proximal femur DEXA scans will also be performed to assess changes in bone mineral density. Triceps skinfold measures and mid upper arm circumference (MUAC) will also be measured to determine corrected arm muscle area (CAMA) [37]. Appetite will be assessed using the Council on Nutrition Appetite Questionnaire (CNAQ) [38] and 24 hour dietary recall recorded using a standardised protocol [39].

**c) Questionnaires: **Assessment of Quality of Life Instrument (AQoL) [40], a four item summary scale created from the CES-D [41] to determine positive affect and a self rated health survey. In addition caregivers will also complete a caregiver burden and strain index [42,43].

#### Additional Information

Monitoring of participants for six months via weekly home visits will allow for an account of any change in accommodation, falls and injuries and any change in health status. At 12 months participants will be contacted via telephone to organise the follow-up assessment. If there is no response then project personnel will contact the next of kin.

#### Economic evaluation

Within study analysis will be undertaken based on observed effects and resource use, including hospital inpatient admission and length of stay from medical records, PBS and MBS use from the Health Insurance Commission (Medicare and Pharmaceutical Benefits), as well as direct costs of the proposed intervention (staff, travel, equipment). Use of community services will also be estimated based on a weekly survey administered to all participants.

### Randomisation and blinding

Participants will be randomly assigned to either the combined nutrition and exercise therapy group or the attention control group following completion of all baseline measures. Group allocation will be managed externally by the Pharmacy Department at the Repatriation General Hospital. Therapists and participants will be aware of treatment allocation however staff performing the outcome assessments and the data analysis will remain unaware of allocation.

### Intervention

Participants allocated to the intervention group will receive a well-integrated, coordinated, individualised care plan combining resistance training and nutrition therapy commencing between day seven to 14 post-surgery and continued over a six month period. Therapists administering the intervention across the two sites will be trained in the established protocols and fidelity to protocols will be addressed through random checks of therapy sessions and fortnightly project staff meetings.

The Otago exercise program [44] has been chosen for its ease of implementation, affordability and effectiveness in terms of falls reduction in older adults [13,14,45]. The program will be supervised until the physiotherapist feels confident that the participant is capable of carrying out the strength, balance and walking program independently and safely. At this point, they will be encouraged to complete the exercise program at a minimum of tri-weekly, along with tri-weekly walking outside of the home with the physiotherapist continuing to visit on a fortnightly basis for the purpose of encouragement, review and upgrading the program. This will all be recorded in an exercise diary maintained by patients and carers to monitor adherence.

As a basis for developing a nutrition program that will achieve energy balance (and hence prevent clinically significant weight loss), the resting energy expenditure (REE) of each participant will be measured using indirect calorimetry. Achieving the shortfall between estimated dietary intake (specifically energy and protein) and measured or recommended requirements, whilst considering risk factors threatening nutritional well-being, will become the focus of the nutrition care plan. Strategies for achieving energy and nutrient requirements will include counselling with attention to timing, size and frequency of meals, recommendations of nutrient dense foods, provision of recipes and referral to community meal programs and providers and supplementation with commercial liquid diets, protein supplements or multi-vitamins where deemed necessary by a dietitian. Intake of any prescribed supplements will be recorded by nursing staff while in hospital, and by the patient or caregiver once discharged home. Dietary intake and requirements will be reviewed on a fortnightly basis by a dietitian and modified as required.

Participants allocated to the attention control group will continue therapy as prescribed during hospital admission (acute and rehabilitation) according to current practice. Social visits of equivalent duration will occur to match the active intervention group. The project physiotherapist and dietitian will provide participants with generic nutrition, exercise and falls information in line with Government recommendations to provide some relevant topics for discussion, this information will also be given to the intervention group.

### Sample Size Considerations

Gait speed at six months was chosen as the primary outcome because safe, independent walking is a high priority amongst patients participating in rehabilitation, and gait speed is often recommended as a measure of status and outcome [46,47]. Using gait speed data collected from a recent study undertaken at the Adelaide site (n = 100) [28] it was calculated that a sample size of 176 participants per group will achieve a statistically and clinically significant 20% difference between the two groups (power 80%, alpha = 0.05). To allow for deaths and withdrawals (30%), 460 participants will be recruited, 230 per group.

### Statistical Analysis

Data will be maintained centrally at Flinders University with double entry of data into SPSS software to verify accuracy. Primary analysis for this study will be undertaken using intention to treat principles. Location of central tendency and distribution will be determined and data presented as appropriate. Independent sample t-tests, Mann-Whitney U tests and Chi-square test of association will be used as appropriate to compare groups at baseline. To determine differences between the groups at the primary end-point (six months), and at 12 month follow up ANCOVA or logistic regression will be used with models adjusted according to potential confounders. The analyses will be undertaken by a statistician blinded to group allocation.

### Time frame

This study has a 3 year time frame. Recruitment commenced in June of 2007, follow up assessment will continue until September 2009, after which time data analysis will be performed and a final report will be drafted.

## Discussion

The INTERACTIVE trial is among the first to combine nutrition and exercise therapy as an intervention to address the serious consequence of rapid deconditioning and weight loss and subsequent ability to regain pre-morbid function in older patients post PFF. It differs from previous studies in this area in a number of significant ways. Commencement of the intervention will occur sooner than any previous work in an attempt to intercept the rapid deconditioning often observed amongst older adults admitted to hospital. The duration of both components of the study intervention will be longer than any previous work in this area, with weekly follow up over a six month period. This is based on evidence to suggest that weight loss continues for at least 12 weeks following PFF [28] and that training improvements achieved during a 12 week exercise program are lost after the program is completed [21].

The benefits of an exercise program to maintain strength and balance and reduce the risk of falls are well established [13-17], however maintaining patient motivation and adherence to a program is an ongoing challenge faced by therapists. The intensity of the follow up in this trial (fortnightly home visits by the physiotherapist) is greater than the Otago program (consisting of four primarily instructive home visits) due to the need for maintaining sufficient challenge in a clinical group that are likely to increase their ability over time and the complexity of pre-existing medical conditions.

The Otago program is yet to be evaluated amongst patients recovering from PFF and provides a pragmatic alternative to other exercise programs due to its established effectiveness, ease of implementation and affordability.

Through regular supervision attempts will be made to maintain patient motivation, continually set realistic and achievable goals and provide an opportunity to address factors affecting adherence. Another strategy targeted at improving adherence is to involve close relatives or immediate caregivers where appropriate. This comprehensive data collection will provide an opportunity to identify groups most likely to respond to such an intervention, which will then inform future service provision. This trial will also be one of the first to utilise body composition measures obtained through DEXA scans to accurately assess for change in muscle mass following such a comprehensive therapy program administered over six months.

Previous studies have failed to consider the need for provision of an adequate amount of energy and protein to address the deficits associated with pre-existing undernutrition, injury and surgical stress and inadequate dietary intakes during recovery from PFF [26]. A comprehensive approach to nutrition therapy combining education, counselling, dietary modification and supplementation differs from previous work, however was deemed necessary given the lack of evidence that oral supplements alone can improve outcomes in this clinical group [26]. If oral supplements are considered appropriate then they will be prescribed as a medication, an approach that has been observed to improve adherence in older adults [48,49].

This study is also unique in that the impact on caregivers and overall costs and resource use will be carefully measured. Measurement of caregiver strain and burden, quality of life and positive affect over time will provide an opportunity to capture whether the potentially negative impact of frequent visits in the home and possible invasion of privacy are outweighed in the long term by the increased independence and quality of life experienced by the PFF sufferer. The comprehensive analysis relating to both direct and indirect costs of the intervention is also unique and will provide realistic data on the feasibility of the intervention becoming an ongoing service. It is important to provide adequate rehabilitation post PFF to return people as close to premorbid function as possible so that physical decline, hospital re-admission and even residential care admission are avoided. The results of this trial will help to guide the development of more effective rehabilitation programs following PFF, which may ultimately lead to reduced health care costs, and improvements in mobility, independence and quality of life for PFF sufferers.

## Competing interests

The author(s) declare that they have no competing interests.

## Authors' contributions

ST and KH are research higher degree students actively involved in implementation of the study. All other authors wrote the grant application for this project that was funded by National Health and Medical Research Council in 2007. The grant application formed the basis for this manuscript which was jointly drafted by ST and KH with all other authors contributing to its critical review and approving the final draft.

## Pre-publication history

The pre-publication history for this paper can be accessed here:


